# The effects of physical exercise on college students’ academic achievement: the chain mediating role of emotional intelligence and academic mood

**DOI:** 10.3389/fpsyg.2025.1642778

**Published:** 2025-08-13

**Authors:** Yong Jiang, Siyu Ren

**Affiliations:** School of Physical Education, Liaoning Normal University, Dalian, China

**Keywords:** physical exercise, academic achievement, emotional intelligence, academic mood, chain mediation

## Abstract

**Introduction:**

This study aims to explore the mechanism of physical exercise’s influence on the academic achievement of contemporary college students, and focuses on the mediating role of emotional intelligence and academic mood in it.

**Methods:**

Through a large-scale questionnaire survey of 540 college students, a chain mediation model was constructed, and the mediating effects of emotional intelligence and academic mood were examined and analyzed using structural equation modeling and Bootstrap method.

**Results:**

(1) Physical exercise can positively predict college students’ academic achievement; (2) Physical exercise can mediate college students’ academic achievement through emotional intelligence and academic mood, respectively; (3) Physical exercise can affect college students’ academic achievement through emotional intelligence and academic mood in a chain. Three pathways of mediating effects: physical exercise → emotional intelligence → academic achievement (path 1), physical exercise → academic mood → academic achievement (path 2), and physical exercise → emotional intelligence → academic mood → academic achievement (path 3).

**Discussion:**

The results of the study show that physical exercise not only directly promotes academic achievement, but also indirectly affects academic achievement through the mediating effect of emotional intelligence and academic mood. This study provides empirical evidence for an in-depth understanding of the relationship between physical exercise and academic achievement, and provides theoretical references for the synergistic promotion of college students’ mental health and academic development.

## 1 Introduction

Nowadays, the development of high-quality education has become a new goal for the development of education in the new era. Academic achievement is an important indicator of the level of quality of education and psychological development of students. Academic achievement reflects a student’s level of learning, which refers to the knowledge and skills a student acquires through specific instruction and training in a specific, relatively limited framework (Nietzel and Bernstein, 1991). With the development of research theories and the advancement of advanced science and technology, the research on physical exercise and academic achievement has made great breakthroughs and aroused extensive attention from the academic community (Zan, 2018). Exploring the effects of physical exercise on college students’ academic achievement has important theoretical significance and practical value.

## 2 Chain mediation hypothesis model of the relationship between physical exercise and academic achievement

### 2.1 The effect of physical exercise on college students’ academic achievement

Academic achievement refers to students’ harvesting of relevant knowledge and skills through stages of learning (Mccombs, 1989), including cognitive and affective gains (Astin and Antonio, 2012). Academic achievement is comprehensive and complex, and we cannot rely solely on academic performance to judge students’ academic achievement, but need to consider students’ performance in the learning process (Wen Fei and Qian Yuan, 2013), including grades, overall quality and other aspects of learning (Ben and Long, 2004), as well as learning outcomes and learning behaviors and attitudes (Yan Fei et al., 2011). Physical exercise is a physical exercise that promotes the healthy development of the body and mind and regulates the spirit through the use of physical exercises of a certain intensity, frequency and duration according to the needs of the body and health (Yu Bao, 2001). Participation in sports not only improves physical and mental health (Yong Feng, 2001), but also enhances cognition, self-esteem, self-confidence and thus academic achievement (Busch et al., 2014). Some studies have shown that moderate physical exercise not only accelerates blood circulation, but also promotes the development of the sensory and nervous systems, which indirectly has a positive impact on academic performance (Cheng and Zhi, 2004). Moreover, physical exercise at a consistent frequency and over a long period of time has a positive impact on students’ academic performance (Davis et al., 2011).

Therefore, the research hypothesis H1: Physical exercise positively predicts college students’ academic achievement is proposed.

### 2.2 Mediating effects of emotional intelligence

One of the concerns of this study regarding mediating mechanisms is the mediating role of emotional intelligence in college students. Emotional intelligence, also known as Emotional Quotient (EQ) (Xue Han, 2023), is a fundamental ability that allows for self-awareness and awareness of the emotions of others (Mayer et al., 1999). It is also a skill that allows you to deeply interpret your own and other people’s emotions, to rationally control impulsive emotions, and to accomplish appropriate management of various interpersonal relationships (Salovey and Mayer, 1990). Emotional intelligence is not a trait that an individual is born with, but rather an ability that an individual acquires and strengthens later in life, and emotional intelligence helps an individual to perceive and deal with internal and external emotional problems (Yuan Li, 2004). Higher levels of emotional intelligence are associated with academic achievement. It can be interpreted that higher emotional intelligence allows students to pursue their interests more vigorously and think more broadly about subjects of interest, and that the development of emotional intelligence can provide educators with significant opportunities to improve academic achievement and emotional competence (Yan Yuan, 2019). Petrides, Frederickson and Furnham found that students with higher emotional intelligence had better attitudes and academic performance. They used the Bar-on Emotional Intelligence Scale to categorize students into A, B, and C. The results of their study found that academic achievement was significantly correlated with emotional intelligence as a whole as well as with several dimensions (Furnham, 2004).

Physical exercise can be used as an emotion regulation strategy, and individuals who take this approach to improving their mood have higher levels of emotional intelligence. Compared with college students who do not regularly participate in physical exercise, college students who regularly participate in sports have more advantages in several dimensions of emotional intelligence such as problem awareness, emotional control, and problem processing, and in the long run, the positive effects of sports on emotional intelligence will be clearly reflected in college students who regularly exercise (Solanki and Lane, 2010). Therefore, regular participation in sports helps college students develop good psychological qualities and promotes emotional intelligence, which in turn improves students’ academic achievement (Yong Jun, 2020).

Therefore, the research hypothesis H2: Physical exercise positively predicts college students’ academic achievement through the mediating role of emotional intelligence is proposed.

### 2.3 Mediating effects of academic mood

Another mediating variable selected for this study was college students’ academic mood. Academic mood refers to all the emotional–emotional experiences that students feel and produce during the learning process (Pekrun et al., 2002). This includes not only the range of academic moods that students experience when they learn of academic success or failure, but also the emotions they experience during classroom learning, daily assignments, and exams (Guo and Yan, 2005). It has been shown that positive exercise-oriented academic mood and positive outcome-oriented academic mood contribute to the academic achievement of college students, while negative exercise-oriented academic mood and negative outcome-oriented academic mood negatively affect the academic achievement of college students. College students who experience more positive academic moods such as happiness, satisfaction, relaxation, and pride in their studies have clear learning goals, affirm their academic value, have a strong sense of identity and belonging to their studies, and devote more time and energy to their studies, and therefore are more likely to achieve outstanding academic achievement (Goetz et al., 2008). Exercise improves moods that contain both high arousal negative emotions like anxiety and low arousal negative emotions like depression, frustration, and sadness. This study focuses specifically on positive academic mood because our research hypothesis centers on the facilitative pathways between physical exercise and academic achievement. Positive academic moods (e.g., enjoyment, hope, pride, and relief) have been shown in the literature to be direct contributors to cognitive resources, motivation to learn, self-regulated learning strategies, and ultimately academic achievement. We aimed to test primarily the mediating role of these positive mood states induced or enhanced by physical exercise. Therefore, physical exercise improves academic mood and thus academic achievement among college students (Zhao Hui, 2020).

Therefore, the research hypothesis H3: Physical exercise positively predicts college students’ academic achievement through the mediating role of academic mood is proposed.

### 2.4 Chain mediation between emotional intelligence and academic mood

Emotional intelligence and academic mood are recognized as important aspects of emotional health (Jia, 2023), and there is a strong and complex link between them. Academic mood has been categorized into positive academic mood and negative academic mood. Increased emotional intelligence can help reduce negative emotions and increase positive emotions (Jia, 2023), and, but groups with higher emotional intelligence tend to have lower levels of anxiety (Dewaele et al., 2010), so emotional intelligence is an important determinant of anxiety or happiness in the classroom environment. Positive emotions can help learners regulate the allocation of cognitive resources, stimulate interest and motivation in learning, adopt flexible and creative learning strategies, and promote self-directed learning, thus enhancing academic performance. Negative emotions, on the other hand, exert a negative influence and hinder academic development. The theory also emphasizes the bi-directional dynamics between academic mood, the learning process, and academic achievement, whereby the assessment of the controllability and value of the learning process and outcomes triggers academic mood, which in turn influences academic exercise and achievement (Yu and Lan, 2024).

Therefore, the research hypothesis H4: Emotional intelligence and academic mood play a chain mediating role in physical exercise and academic achievement is proposed.

**Figure 1:**
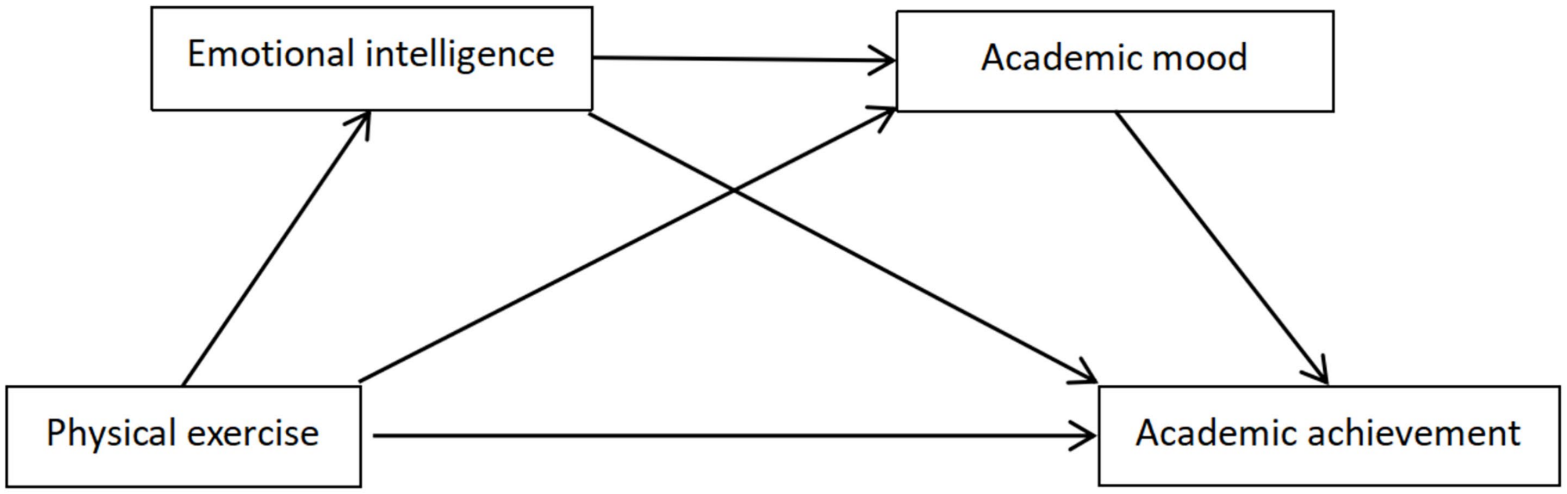
Hypothesized model of physical exercise on academic achievement of college students.

As a result, the research hypothesis model was constructed as shown in Figure 1.

## 3 Research objects and methods

### 3.1 Objects of study

The research object of this article is the mediating role of physical exercise on academic achievement, emotional intelligence, and academic mood of college students. In order to improve the representativeness of the sample to the whole and to ensure the coverage of the group of college students at different stages of study, the study was based on grade level and used stratified random sampling to select four grades of college students as the subjects, with the lower grades as the main research object, and a total of 580 questionnaires were distributed. Of these, 40 invalid questionnaires were excluded, consisting of extreme responses, incomplete responses, and responses that did not meet the time limit, with extreme responses being those that were completely consistent and overly biased. 540 valid questionnaires were recovered, with an effective recovery rate of 93.1%, and the sample size met the criteria (see Table 1).

**Table 1:** Sample demographics.

Variable	Categories	Numbers	Percentage
Gender	Male	306	56.7%
	Female	234	43.3%
Grade	First-year university student	475	88%
	Second-year university student	11	2%
	Third-year university student	24	4.4%
	Fourth-year university student	30	5.6%

Although variables such as major type, basic academic level, and socioeconomic status may influence the relationships examined, these covariates were not controlled in the current analytical model due to sampling constraints. Future studies should incorporate these factors to enhance the robustness of findings, particularly given potential differences in emotional regulation demands across disciplines.

### 3.2 Measurement

#### 3.2.1 Physical exercise rating scale

The Physical Exercise Rating Scale compiled by a Japanese scholar, Mr. Hashimoto, and revised by a Chinese scholar, Mr. Liang Deqing, was used to measure the physical exercise rating from the three dimensions of intensity, time, and frequency of participation in physical exercise in order to calculate the amount of physical exercise, and the higher the calculated score, the greater the amount of physical exercise of the individual.

#### 3.2.2 Academic achievement rating scale

Using Lu and Wei, 2016 academic performance measurement standards for college students, there are a total of 18 questions, including professional ethics, core competencies, and citizenship. The questionnaire was rated on a 5-point scale, with higher scores indicating higher levels of academic achievement, and the Cronbach’s alpha coefficient for this measure was 0.960.

#### 3.2.3 Emotional intelligence scale

Selected from the Self-Stated Emotional Intelligence Scale developed by Schutte in 1998 based on Mayer and Salovey’s theory of emotional intelligence. The scale consists of 33 questions with 4 dimensions, including emotion perception, self-emotion regulation, others’ emotion regulation and emotion utilization; the scale is scored in the form of a 5-point Likert scale, with 5 points for each question out of a total of 165 points, of which the 5th, 28th, and 33rd questions are reverse-scored questions. Higher scores indicate higher levels of emotional intelligence and vice versa, and the Cronbach’s alpha coefficient of this measurement questionnaire is 0.945.

#### 3.2.4 Academic mood scale

The Academic Mood Scale for College Students compiled by Xu Xiancai was used to select the positive academic mood section with 22 questions, including two dimensions of positive exercise-oriented mood and positive outcome-oriented mood, and the Cronbach’s alpha coefficient of this measurement questionnaire was 0.924.

### 3.3 Statistical processing

This study used SPSS 29.0 statistical software for data analysis. First, test reliability was tested using Cronbach’s alpha test and Harman’s one-way test was used to test for common method bias after data collection. Second, after importing the data into SPSS, demographic analysis was performed using descriptive statistics. Pearson correlation coefficients were used to analyze the correlations between physical exercise, academic achievement, and emotional intelligence with academic mood. The study was analyzed by performing multiple regression analysis using Model 6 in the PROCESS macro program and evaluating the significance level of the mediating effect using the Bootstrap test.

## 4 Results

### 4.1 Control and testing of common method deviations

Since all the data in this study came from subjective questionnaires, a common method bias test was needed. The common method bias test was conducted by using the Harman one-way test, and the test results showed that the number of factors with eigenvalues greater than 1 reached 12, and the maximum factor variance explained rate was 36.590%, which was lower than the 40% critical criterion, indicating that the data collected in this study did not have significant common method bias, and met the statistical requirements.

### 4.2 Independent sample *t*-test

Independent samples *t*-test was used to analyze the differences in physical exercise, academic achievement, emotional intelligence and academic mood among college students of different genders, as shown in Table 2. Significant gender differences were found on all core variables: physical exercise level (*t* = 4.632, *p* < 0.001), academic achievement (*t* = 2.402, *p* = 0.017), emotional intelligence (*t* = 2.936, *p* = 0.003), and academic mood (*t* = 3.366, *p* < 0.001) were higher in boys than in girls. These findings should be interpreted from a sociocultural perspective, as men’s participation in team sports (e.g., basketball, soccer) is significantly higher in the Chinese university environment. This cultural pattern likely amplifies the male advantage in “physical exercise” scores. Rather than attributing it to biological determinism.

**Table 2:** Differences in physical exercise, academic achievement, emotional intelligence, and academic mood by gender.

Relevant variable	Gender	N	M ± SD	*t*	*p*
Physical exercise	Male	306	45.856 ± 20.965	4.632	<0.001
	Female	234	37.871 ± 19.020		
Academic achievement	Male	306	3.979 ± 0.702	2.402	0.017
	Female	234	3.832 ± 0.704		
Emotional intelligence	Male	306	3.780 ± 0.558	2.936	0.003
	Female	234	3.639 ± 0.555		
Academic mood	Male	306	3.785 ± 0.670	3.366	<0.001
	Female	234	3.591 ± 0.651		

### 4.3 ANOVA one-way analysis of variance

ANOVA one-way analysis was used to analyze the differences in physical exercise, academic achievement, emotional intelligence and academic mood among college students of different grades. The results showed that freshmen and junior and senior students exhibited significant differences in physical exercise dimensions, as shown in Tables 3–7.

**Table 3:** ANOVA one-way analysis of variance.

Relevant variable	Grade	M ± SD	*F*	*p*
Physical exercise	1 (475)	43.933 ± 19.969	8.231	<0.001
	2 (11)	25.818 ± 17.988		
	3 (24)	31.708 ± 19.741		
	4 (30)	32.700 ± 18.073		
Academic achievement	1 (475)	3.951 ± 0.690	4.310	0.005
	2 (11)	3.434 ± 0.592		
	3 (24)	3.579 ± 0.668		
	4 (30)	3.793 ± 0.886		
Emotional intelligence	1 (475)	3.747 ± 0.538	4.446	0.004
	2 (11)	3.333 ± 0.496		
	3 (24)	3.439 ± 0.403		
	4 (30)	3.633 ± 0.859		
Academic mood	1 (475)	3.743 ± 0.638	7.865	<0.001
	2 (11)	3.058 ± 0.762		
	3 (24)	3.263 ± 0.728		
	4 (30)	3.627 ± 0.820		

**Table 4:** Multiple comparisons of physical exercise among college students of different grades.

Grade	First-year university student (43.933)	Second-year university student (25.818)	Third-year university student (31.708)
Fourth-year university student(32.700)	11.23263*	−6.88182	−0.99167
Third-year university student (31.708)	12.22430*	−5.89015	
Second-year university student (25.818)	18.11445*		

*p < 0.05.

**Table 5:** Multiple comparisons of academic achievement among college students in different years of study.

Grade	First-year university student (3.951)	Second-year university student (3.434)	Third-year university student (3.579)
Fourth-year university student (3.793)	0.15852	−0.35825	−0.21389
Third-year university student (3.579)	0.37241*	−0.14436	
Second-year university student (3.434)	0.51677*		

*p < 0.05.

**Table 6:** Multiple comparisons of emotional intelligence in college students of different years of study.

Grade	First-year university student (3.747)	Second-year university student (3.333)	Third-year university student (3.439)
Fourth-year university student (3.633)	0.11359	−0.3	−0.19394
Third-year university student (3.439)	0.30753*	−0.10606	
Second-year university student (3.333)	0.41359		

*p < 0.05.

**Table 7:** Multiple comparisons of academic mood among college students at different grade levels.

Grade	First-year university student (3.743)	Second-year university student (3.058)	Third-year university student (3.263)
Fourth-year university student (3.627)	0.11579	−0.56942*	−0.36402*
Third-year university student (3.263)	0.47980*	−0.20541	
Second-year university student (3.058)	0.68521*		

*p < 0.05.

### 4.4 Correlation analysis between variables

Pearson correlation coefficient was used to analyze the correlation of the main variables through SPSS29.0, and the results of the test are shown in Table 8, which shows that academic achievement is significantly and positively correlated with physical exercise; emotional intelligence and academic mood are significantly and positively correlated; and physical exercise is significantly and positively correlated with emotional intelligence and academic mood, that is to say, there is a statistically significant positive correlation between the four variables, namely, physical exercise, emotional intelligence, academic mood and academic achievement. The test results show that as the amount of physical exercise increases, the better the academic mood, the higher the level of emotional intelligence, and the stronger the academic achievement of college students.

**Table 8:** Pearson correlation coefficient.

Relevant variable	Physical exercise	Academic achievement	Emotional intelligence	Academic mood
Physical exercise	1			
Academic achievement	0.585**	1		
Emotional intelligence	0.631**	0.660**	1	
Academic mood	0.649**	0.749**	0.690**	1

**p < 0.001.

### 4.5 Mediating effects test

The chain mediating effects of emotional intelligence and academic mood between physical exercise and academic achievement were tested and the results are shown in Table 9. The specific steps are: in the first step, academic achievement was used as the dependent variable, and gender and grade level were entered to control for the possible effects of these variables on academic achievement. In the second step, physical exercise was entered to establish a regression model to examine the total effect of physical exercise on academic achievement after controlling for the variables, and it was found that physical exercise significantly and positively predicted academic achievement (*β* = 0.587, *p* < 0.001), indicating that research hypothesis H1 was valid. In the third step, emotional intelligence and academic mood variables were sequentially added to the model to test whether they were mediating variables between physical exercise and academic achievement and whether there was a chain mediating effect, and it was found that physical exercise was able to significantly and positively predict academic achievement (*β* = 0.097, *p* < 0.05), emotional intelligence was able to positively predict academic achievement (*β* = 0.240, *p* < 0.001), and academic mood positively predicted academic achievement (*β* = 0.524, *p* < 0.001). Also in the test, it was found that physical exercise significantly and positively predicted emotional intelligence (*β* = 0.632, *p* < 0.001) and academic mood (*β* = 0.350, *p* < 0.001), and emotional intelligence positively predicted academic mood (*β* = 0.467, *p* < 0.001). As seen from the above regression coefficients, there was a significant chain mediation between physical exercise and academic achievement for emotional intelligence and academic mood, proving that research hypotheses H2 and H3 were valid.

**Table 9:** Regression analysis between variables.

Equation of regression		Overall fit index			Significance of regression coefficient		
Result variable	Variable of prediction	*R*	*R*2	*F*	*β*	*t*	*p*
Emotional intelligence	Physical exercise	0.631	0.399	118.422	0.632	18.338	0.000
	Gender				−0.005	−0.131	0.896
	Grade				0.010	0.293	0.770
Academic mood	Physical exercise	0.743	0.552	164.897	0.350	9.212	0.000
	Emotional intelligence				0.467	12.513	0.000
	Gender				−0.015	−0.496	0.621
	Grade				−0.005	−0.155	0.877
Academic achievement	Physical exercise	0.778	0.606	164.075	0.097	2.533	0.012
	Emotional intelligence				0.240	6.010	0.000
	Academic mood				0.524	12.895	0.000
	Gender				0.023	0.816	0.415
	Grade				−0.007	−0.261	0.795
Academic achievement	Physical exercise	0.585	0.342	92.936	0.587	16.267	0.000
	Gender				0.013	0.358	0.721
	Grade				−0.005	−0.134	0.894

“Overall fit index” includes *R* (correlation), *R2* (variance explained), *F* (model significance test). “Significance of regression coefficients” has *β* (effect size), *t* (individual predictor test), *p* (*p* < 0.05 indicates significant association).

**Figure 2:**
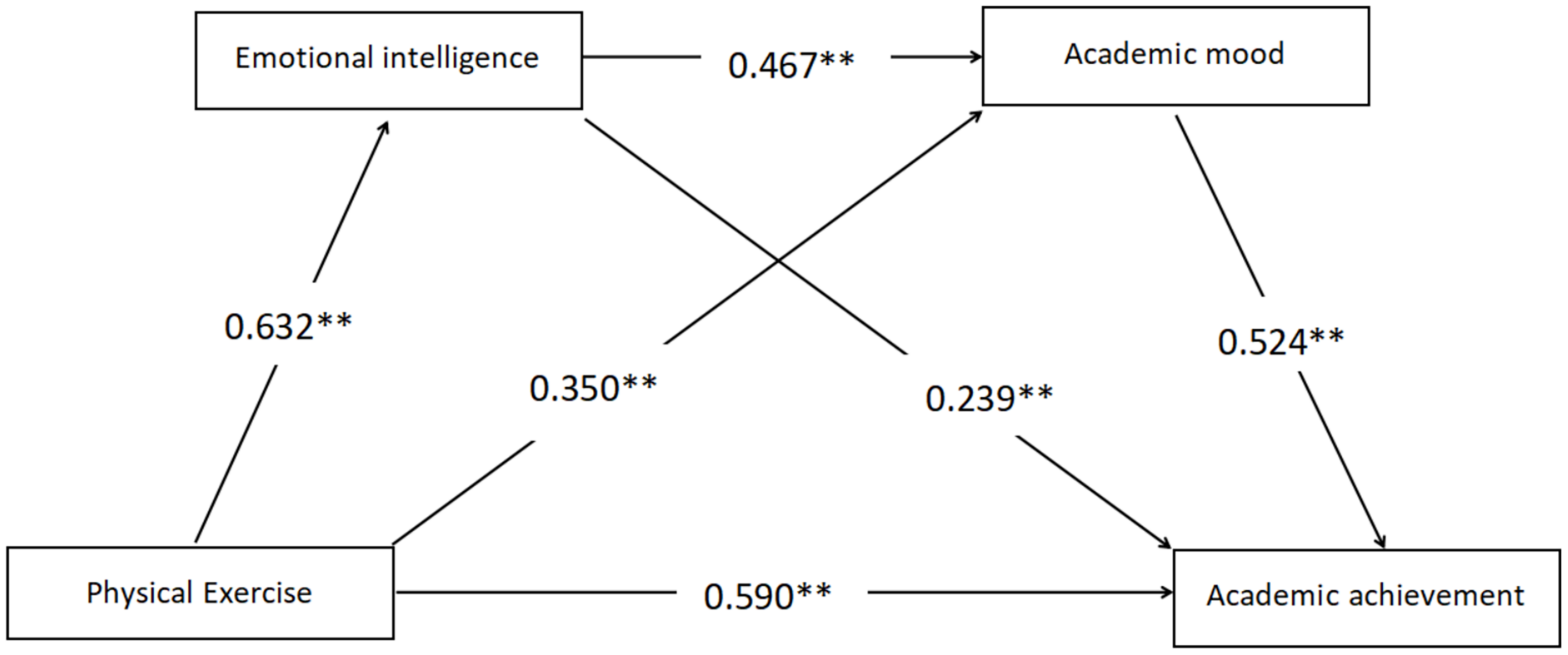
A mediating model of physical exercise affecting students’ academic achievement. **p < 0.01; ***p < 0.001.

Bootstrap test was used to repeat the sampling 5,000 times to test the mediating effects of emotional intelligence and academic mood between physical exercise and academic achievement and the confidence intervals, respectively, as shown in Table 10. The results showed that physical exercise produced a total effect value of 0.0205 on academic achievement, and the 95% confidence intervals for the mediating effects of emotional intelligence and academic mood did not contain 0 (LLCL = 0.0180, ULCL = 0.0229), which indicated that the total effect of physical exercise on academic achievement as well as the mediating effects of the two variables were significant. The value of the direct effect of physical exercise on academic achievement is 0.0034 (direct path), Bootstrap 95% confidence interval does not contain 0 (LLCL = 0.0008, ULCL = 0.0060), which indicates that there is a significant direct effect of physical exercise on academic achievement, with an effect value of 16.59% of the total effect. Based on the above results, the following model diagram is derived as shown in Figure 2.

**Table 10:** Proportion of the mediating effect.

Effect	Paths	Effect value	Standard error	LLCL	ULCL	Effect ratio
Total effect		0.0205	0.0013	0.0180	0.0229	100%
Direct effect	Direct path	0.0034	0.0013	0.0008	0.0060	16.59%
Total indirect effect		0.0171	0.0013	0.0145	0.0195	83.41%
Indirect effect	Ind1	0.0053	0.0013	0.0029	0.0077	25.85%
	Ind2	0.0064	0.0010	0.0045	0.0085	31.22%
	Ind3	0.0054	0.0008	0.0040	0.0070	26.34%

## 5 Discussion

### 5.1 The relationship between physical exercise and academic achievement

The results of the study found a significant positive correlation between physical exercise and academic achievement of college students, which verified hypothesis H1. And the positive predictive effect of physical exercise and college students’ academic achievement remained significant after the inclusion of mediating variables. How to enhance the physical fitness of students and improve the enthusiasm of sports have become a top priority, and only in this way can we more quickly promote the theory of sports for all and a strong sports country. Research has shown that physical exercise improves students’ intelligence, which is reflected in increased academic achievement (Graham, 2010; Harmon, 2013). Physical health is the foundation of academic development, and good physical condition can ensure that students maintain abundant energy and sustained attention, thus guaranteeing efficient learning. In addition, physical exercise has a significant effect on students’ mental health. By participating in sports, students can effectively release pressure and relieve anxiety, and then maintain a good psychological state. This positive psychological state is an important support for academic success and helps students to better cope with the challenges and difficulties in the learning process. To summarize, physical exercise has a significant effect on the improvement of academic achievement. Integrating physical exercise into daily life can not only inject continuous power for knowledge accumulation, ability improvement and character building, but also lead students to realize the goals of comprehensive development and academic excellence.

### 5.2 Mediating role of emotional intelligence in physical exercise and academic achievement

It was found that the effect of physical exercise on academic achievement can also be realized through an indirect pathway, emotional intelligence, validating hypothesis H2. The predictive effect of emotional intelligence on academic achievement is consistent with the findings of previous studies. Emotional intelligence is a predictor of academic achievement (Shu Ting, 2006). Emotional intelligence has a direct impact on the mental health of college students. Students with higher levels of emotional intelligence are more likely to be able to feel and control their personal emotions, maintain good interpersonal relationships with others, and be resilient to stress (Baron and Parker, 2002), Physical exercise is a key way to promote the development of emotional intelligence. Studies have shown that sustained participation in physical exercise helps college students improve their psychological quality and significantly increases their emotional intelligence. If the mediating effect of emotional intelligence between physical exercise and academic achievement is ignored, it is difficult to maximize the effect of physical exercise on academic achievement. Therefore, paying attention to the cultivation of emotional intelligence is a necessary condition to realize the synergistic development of physical exercise and academic achievement. Thus, it can be concluded that physical exercise promotes the progress of college students’ academic achievement by enhancing their emotional intelligence.

### 5.3 The mediating role of academic mood in physical exercise and academic achievement

It was also found that the effect of physical exercise on academic achievement can also occur indirectly on academic achievement by promoting academic mood, testing hypothesis H3. The predictive effect of academic mood on academic achievement is consistent with the findings of previous studies. There is a correlation between the amount of physical exercise and the physical and mental health of students. Many research studies have shown that students who spend more time playing sports have lower psychological stress (De Qing, 1994). While participating in physical exercise, students are able to release stress and regulate their moods, thereby enhancing levels of positive academic mood. Physical exercise significantly improves students’ mental health, which in turn has a positive impact on academic mood. Increased academic initiative, improved academic focus, and higher academic achievement in a positive academic mood. Thus, research suggests that students’ academic achievement can be promoted through the proximal psychological process of promoting positive academic mood and suppressing negative academic mood. Academic mood serves as a mediating variable that transmits and amplifies the positive effects of physical exercise on academic achievement.

### 5.4 Chain mediation between emotional intelligence and academic mood

It was also found that emotional intelligence and academic mood, in addition to their respective individual mediating roles between physical exercise and academic achievement, both could also chain mediate between physical exercise and academic achievement, validating hypothesis H4. Emotional intelligence is the ability of an individual to recognize, understand, express, and regulate his or her own and others’ emotions. Individuals with high levels of emotional intelligence are able to recognize, appraise, and regulate negative emotions, as well as generate and use positive emotions to promote thinking (Ciarrochi and Mayer, 2007). This ability not only directly affects an individual’s emotional state, but also influences academic achievement by regulating academic mood. The chained mediation of emotional intelligence and academic mood is important in an individual’s academic achievement. By increasing emotional intelligence, individuals are better able to manage their emotions, which in turn enhances positive academic mood and promotes academic achievement.

### 5.5 Practical implications and intervention strategies

As shown in Table 10, the Ind1 effect for Emotional Intelligence was 25.85%, and physical exercise developed self-awareness and interpersonal skills, which directly improved cognitive regulation. The Ind2 effect for academic mood was 31.22%, and exercise-induced positive emotions directly increased learning engagement. The Ind3 effect of the Emotional Intelligence to Academic mood pathway was 26.34%, with Emotional Intelligence enabling students to transform emotional competence into sustained academic motivation, thus amplifying the impact of academic mood. In conclusion, depending on the effect size, a phased and integrated intervention strategy is recommended: (1) Promotion of physical exercise as a foundation. (2) Simultaneously develop emotional intelligence and provide students with enduring self-regulation skills. (3) Implement immediate academic mood support that directly enhances learning experiences and outcomes. (4) Explicitly integrating emotional intelligence training with academic mood management by teaching students how to use emotional intelligence skills to cultivate positive academic moods and counteract negative academic moods, thereby maximizing the synergistic chain effect revealed in this study. Universities should design programs that fully integrate these elements into their student support services and wellness initiatives.

## 6 Conclusion

The findings primarily apply to lower-grade undergraduates (freshmen accounted for 88% of the sample), as the limited representation of higher-grade students may affect the generalizability of results to senior cohorts. This study expands the field of research on academic achievement by introducing “academic achievement” into the study of school sports. Conclusions were drawn: (1) There is a strong relationship between physical exercise, emotional intelligence, academic mood, and academic achievement among college students. (2) Physical exercise is an important influence on college students’ academic achievement, and can also have an indirect effect on academic achievement through the pathways of emotional intelligence and academic mood.

From the perspective of school sports, it explores a possible solution path to enhance college students’ academic achievement, and also provides empirical support for how colleges and universities can actively support college students to participate in physical exercise and enhance their academic achievement. In the context of the reality of vigorously improving the quality of higher education, it is important to cultivate lifelong sports awareness among students, promote their physical and mental health, and effectively ensure the quality of their studies.

## 7 Limitations and future directions

There are several limitations to this study. First, the sample is overrepresented by freshmen (88%), which may limit the extrapolation of the results to upperclassmen. Second, not controlling for key covariates such as major type, basic academic level, socioeconomic status may confound the observed relationships. Nevertheless, the results of this study highlight the importance of systematically integrating physical exercise into the higher education system, which goes far beyond “fostering a lifelong awareness of exercise.” To effectively leverage the contribution of physical exercise to academic achievement, colleges and universities need to move beyond generalized advocacy and begin to build a framework for actionable implementation programs: Integrate physical exercise scientifically and structurally into the curriculum, and design diversified activities to cover different interests and abilities; Provide evidence-based exercise instruction to help students understand the positive physical, mental, and academic effects of exercise of appropriate intensity, frequency, and duration; As well as optimizing campus sports resources and environment to create convenient and friendly sports conditions. Future research should use a stratified sampling strategy across grades and disciplines and integrate the covariates mentioned above to more clearly elucidate the mechanisms by which physical exercise affects academic achievement and to enhance the robustness of the findings. Through a combination of specific institutionalized initiatives in higher education and more rigorous research in the future, it is possible to synergistically promote the win-win goal of students’ physical and mental health and academic development, and to provide practical support for improving the quality of higher education.

## Data Availability

The original contributions presented in the study are included in the article/Supplementary material, further inquiries can be directed to the corresponding author.
